# Access to artemisinin-based combination therapy (ACT) and quinine in malaria holoendemic regions of western Kenya

**DOI:** 10.1186/1475-2875-13-290

**Published:** 2014-07-28

**Authors:** Carren A Watsierah, Collins Ouma

**Affiliations:** 1 Department of Public Health, Maseno University, Private Bag, Maseno, Kenya; 2 Department of Biomedical Sciences and Technology, Maseno University, Private Bag, Maseno, Kenya

## Abstract

**Background:**

Artemisinin-based combination therapy (ACT) has been adopted as the most effective treatment against malaria in many endemic countries like Kenya while quinine has remained the second line. The objective of the current study was to assess access to Kenya’s policy recommended anti-malarials, ACT and quinine in the public, private and not-for-profit drug outlets in western Kenya.

**Methods:**

A cross-sectional survey using purposive sampling of 288 outlets (126 public, 96 private, 66 not-for-profit) was conducted in western Kenya in two regions with varying *Plasmodium falciparum* endemicities. Information on access (availability, price, affordability) on ACT and quinine was collected using the WHO and Healthcare Associated Infection (HAI) standardized methodologies for availability, prices and affordability of drugs. From a Ministry of Health database, the following were included in the analyses: one (1) main public hospital, followed by random selection of five hospitals under this main facility. Eight other public outlets under each of the hospitals were selected, to a total of 96. Matching number of private outlets (n = 96), all (66) not-for-profit outlets and additional 30 public health facilities were sampled to get the required sample size of 288.

**Results:**

More public 111 (88.1%) and not-for-profit 27 (40.9%) outlets stocked subsidized ACT (artemether-lumefantrine, AL). Other artemisinin-based combinations were widely available for both children 93 (96.9%) and adults 82 (85.0%) in private outlets. Frequent stock-outs were in public in 106 (84%), reporting three times or more stock-outs in three months. Subsidized ACT (AL) was sold at median price of USD 0.94 and 0.75 in private and not-for-profit outlets respectively. The costs was higher than recommended price of USD 0.5 and requiring up to 0.20-0.25 days of disposable income for households in lowest economic status.

**Conclusion:**

There is low availability of subsidized ACT (AL) and higher frequency of stock-outs in government facilities, while private sector sells AL at higher prices, thus making it less affordable to many households. These factors determine the adherence to the dosing schedules during the treatment course and thus the evaluation of the subsidy policy, its implementation and role in malaria burden in this region is compulsory.

## Background

Malaria still poses a great burden in many parts of Africa despite the availability of many interventions focused on preventive and therapeutic approaches [1]. Artemisinin-based combination therapy (ACT) has been adopted as the most effective treatment option against malaria in many countries following the widespread malarial parasite resistance to more affordable anti-malarial drugs, such as chloroquine and sulphadoxine-pyrimethamine (SP) [2]. Kenya adopted the new malaria policy in 2004, which recommends the use of artemether 20 mg-lumefantrine 120 mg (AL) as the first-line drug for treatment of uncomplicated malaria [3], while quinine is preferred for complicated and severe malaria.

The World Health Organization (WHO) however, stresses the fact that many strategies to increase access to effective anti-malarial drugs have been put in place, but in the year 2009, less than 15% of children under-five years of age received ACT when they presented with fever in 11 out of 13 African countries [4]. In addition, most mortality in children who are able to attend a health facility with adequate staff and supplies occurs within 24 hours after admission [5]. Consequently, it is evident that most mortality occurring in such set-ups are as a result of delay in home management with effective drugs, thus underscoring the importance of early treatment for preventing such mortalities [5]. It is, therefore, critical to improve access to recommended drugs. One way of approaching this problem is to ensure access to treatment using effective anti-malarials like ACT and quinine which could be given promptly even at primary health care facilities as highlighted by WHO, to ensure access and minimize parasite resistance [5].

Even with the efforts to improve the case management of malaria, the success of policy implementation and effectiveness is measured by the availability of the recommended drugs at the point of care [6]. Many patients in Africa use private sector as their primary source of medicines, with 50% of febrile cases reported to be treated in this sector [7], despite the fact that the manufacturer’s selling prices and final patient prices range from 56% to 358%, making treatment unaffordable [8]. On average, households in many malaria endemic countries spend up to 90% of their household expenditure on medicines, portraying high anti-malarial pricing as an important contributing factor to the lack of access to ACT [9]. Furthermore, a recent study [10] demonstrated that there were huge increases in quality-assured ACT (QAACT) availability (25.8—51.9% points), and market share (15.9—40.3% points), driven mainly by changes in the private for-profit sector. The same study observed that large falls in median price for QAACTs per adult equivalent dose was seen in the private for-profit sector in six pilots, ranging from US$1.28 to $4.82. The recent findings provide additional evidence that anti-malarial pricing is a critical factor in access to ACT in sub-Saharan African countries.

To enhance ACT policy implementation and further proper management of malaria, Kenya government has scaled up case management strategies as follows: 1) all public health institutions offer ACT free-of-charge; 2) introducing cheaper artemether-lumefantrine (AL) to be sold at Kenyan shillings 40 (USD 0.5) in retail drug outlets [11]; and 3) children who are below 13 years of age are treated for free in public health facilities. Despite the efforts to improve access, ACT remains registered as prescription-only drug recommended for formal sector providers [12] and, in 2010, the treatment policy was universally changed to include the use of ACT for only laboratory-confirmed malaria cases [13,14]. The two regulations hence limit access of households to these drugs further causing delay in treatment for more than 24 hours. However, access to ACT at the point-of-care and at the subsidized prices at any given time, especially in malaria endemic areas, with high levels (≥40%) of malaria prevalence throughout the year remains unknown [11]. Frequent stock-outs have been reported in a few studies as leading to the use of several inappropriate anti-malarials in the households [15-18]. Availability of a particular drug in the market is an important factor in determining which anti-malarials are bought for use [19] of which, in addition to high cost of anti-malarial, are important contributing factors to lack of access [20]. Furthermore, two years following the Kenyan government rolling-out the subsidy policy on ACT, namely artemether 20 mg-lumefantrine 120 mg (AL) as the first-line treatment, and quinine still being the preferred treatment of severe malaria, no survey has been carried out to date to evaluate availability of anti-malarials in malaria-prone regions. Most of the studies reporting ACT availability before or immediately after the roll-out were based on country-wide surveys and results were not attributed to fit the differences in regional prevalence of malaria.

It is on this background that the current study assessed access to Kenya’s policy recommended anti-malarials, ACT and quinine in the public, private and not-for-profit drug outlets using the WHO and Healthcare Associated Infection (HAI) standardized methodologies for availability, prices and affordability of drugs.

## Methods

### Study design and study area

A cross-sectional study was conducted between February and May 2012 in Nyanza Province in western Kenya. Two regions, endemic for *Plasmodium falciparum* transmission, but with different levels of risk for malaria in the province, were targeted: the lowlands of the Province (Kisumu, Siaya and Bondo regions) around Lake Victoria with a holoendemic and stable *P. falciparum* transmission (altitude 0–1,300 metres) and Kisii highlands (Kisii, Gucha and Nyamira regions), with an epidemic transmission (>1,300-1,750 metres) [21].

Malaria in the region accounts for 40% of out-patient visits and 40% of hospital in-patient admissions, with between 10–15 paediatric cases of severe malaria often complicated with anaemia and malnutrition, on a daily basis [22]. Malaria transmission occurs all year round, peaking in the rainy season months of April and May. The highlands areas suffer epidemic levels, where temperature increases and rainfall variation impacts on vector breeding and malaria transmission with some areas experiencing a prevalence of up to 20% [23].

### Selection of outlets and households

Three-stage sampling approach was used to select drug outlets. For each region, a list of public medical facilities was compiled using a database obtained from the Kenyan Ministry of Health [24]. First, from each of the two selected study areas, the main public hospital was selected, and then five hospitals under the main facility were randomly sampled. Lastly, eight other public outlets under each of the hospitals were selected, to a total of 96. Matching number of private outlets was randomly selected. All (66) not-for-profit outlets and additional 30 public health facilities were sampled to get the required sample size of 288. For every outlet sampled, one household served by the outlet was sampled.

### Data collection

Data was collected through drug auditing and interviews by enumerators who visited the outlets as in pairs. Focus was on tablet anti-malarials only – typically, since this is the most common formulation. Prior to data collection, enumerators who had nursing qualification were trained on the use of the tools prior to pilot testing in nine of each outlet type with regular supervision. Drug outlets were recruited for the survey. In the outlets, providers who consented to participate were asked two screening questions to determine whether (1) the outlet had stocked ACT or quinine, within the previous three months, and (2) if ACT or quinine were available on the day of the survey. All providers answering “yes” to at least one of these questions gave information about the ACT and quinine after informed consent was obtained from them. In outlets with more than one attendant, the provider who was serving the clients at the time of the survey provided the information.

The information collected in the drug outlets included brand name, amount in stock and the frequency of stock out in the last three months, retail price, and dosage form as well as information on packs per weight group. The price of anti-malarials was originally collected in Kenya Shillings (KES), and then later converted to US dollars (USD) for international standardization in costs.

### Data management and statistical analysis

Data collected was checked in the field and at the end of each day cleaned to ensure completeness, consistency, credibility and eligibility. Information captured in audit sheets and interview guide on availability, affordability and prices of anti-malarials as well as their use in households was coded and entered into Statistical Package for Social Sciences (SPSS) version 19. Analysis adopted the WHO and Healthcare Associated Infection (HAI) standardized methodologies for availability, prices and affordability of drugs. Data from the outlets were grouped into three categories and analyzed separately into: 1) public outlets, 2) private outlets and 3) not-for-profit outlets. Furthermore, anti-malarials were stratified as the government subsidized ACT (AL), other formulations of ACT and quinine. Using the mean and median, drug access (price, availability and affordability) was established. For the availability analysis, different dose strengths of the same drug were combined to calculate the overall availability of that particular anti-malarial on the day of the survey.

The median prices of anti-malarials were calculated for each anti-malarial category identified. For comparison, the median price ratios (MPR) of the ACT median price to the current government recommended price for a single treatment course with AL was computed in private and not-for-profit outlets at USD 0.5 (KES 40). For quinine, the MPR of private to not-for-profit outlets was calculated, since there was no government-subsidized price at the moment.

Affordability was expressed as the number of days households with lowest daily disposable income level at KES 150,USD 1.875 [16] would need to work in order to pay for the full adult dose of treatment course with AL, other artemisinin-based combinations and quinine available in the outlet. Chi-square analyses were used for proportionality. Statistical significance was assessed at a *p* ≤ 0.05.

## Results

### Availability of anti-malarials

All outlets stocked more than one category of anti-malarial. More public 111 (88.1%) and not-for-profit outlets 27 (40.9%) surveyed stocked AL, the recommended first-line treatment for uncomplicated malaria. However, less than a third of the private outlets 27 (28.1%) had stocked AL (Table 1). Other forms of ACT were stocked mostly by private outlets 87 (90.6%) and less by not-for profit 8 (12%), while public outlets did not stock any other ACT. All outlets stocked both first-line subsidized ACT (AL) and second-line (quinine) anti-malarials in varied proportions (Table 1).

**Table 1:** Availability of subsidized ACT (AL), other forms of ACT and quinine in the three outlet types

	**Outlet type**		
	**Public n = 126 (%)**	**Private n = 96 (%)**	**Not-for-profit n = 66 (%)**
***Availability of anti-malarial drugs***			
**Subsidized ACT (AL)**	111 (88.1)	27 (28.1)	27 (40.9)
**Other ACT**	0 (0)	87 (90.6)	8 (12.0)
**Quinine**	59 (46.8)	68 (70.8)	36 (54.5)

ACT, artemisinin-based combination therapy, AL, artemether-lumefantrine.

### Availability of packs per weight group of subsidized ACT, other forms of ACT and quinine tablet formulation

The subsidized ACT was available in all the four weight-specific presentations in less than half of the total outlets 88 (30.6%), while quinine was available in 137 (47.6%) outlets. Other forms of ACT were widely available for both children 93 (96.9%) and adults 82 (85.0%) in private outlets. The availability varied with the outlet type as shown in Table 2. Availability of packs for different weight groups was also evaluated for AL in the three outlet types. The six tablet pack was in stock in more public outlets 47 (37.3%) than in private 21 (21.9%) and not-for profit 16 (24.2%) outlets (Table 2). The same trend was observed for the twelve AL tablet packs, however, the eighteen pack was in stock in public 66 (52.4%) and not-for-profit 33 (50.0) than in private 12 (12.5%) outlets. Apparently, a higher proportion of quinine tablet formulation was available in private 46 (47.9%) than in public 44 (34.9%) and not-for-profit 28 (42.4%) outlets (Table 2).

**Table 2:** Availability of packs per weight group of subsidized ACT (AL)

	**Outlet type**		
	**Public n = 126 (%)**	**Private n = 96 (%)**	**Not-for-profit n = 66 (%)**
**All the four AL weight specific packs**	53 (42.1)	23 (23.9)	12 (18.2)
**AL 6 tablet pack for 5-14 kg**	47 (37.3)	21 (21.9)	16 (24.2)
**AL 12 tablet pack for15-24 kg**	62 (49.2)	25 (26.0)	12 (18.1)
**AL 18 tablet pack for 25-34 kg**	66 (52.4)	12 (12.5)	33 (50.0)
**AL 24 tablet pack for ≥35 kg**	111 (88.1)	42 (43.8)	46 (69.7)
**Other forms of ACT for children**	0	93 (96.9)	8 (12.1)
**Other forms of ACT for adults**	0	82 (85.0)	9 (13.6)
**Quinine (tablet formulation only)**	44 (34.9)	46 (47.9)	28 (42.4)

ACT, Artemisinin-based Combination Therapy, AL, Artemether-Lumefantrine.

### Stock-out of anti-malarials

Stock-out in the past three months was defined as absence of drugs from stock for at least seven consecutive days [25]. The stock-out rates in public outlets was most frequent in the last three months with 106 (84.0%) outlets reporting three times or more stock-outs. On the other hand, private sector suffered stock-outs once in three months in 70 (73.0%) outlets while in not-for-profit outlets, there was no stock-out in 57 (87.0%) in the past three months.

The decision for restocking a particular anti-malarial was mainly mediated by the recommendation by the government in 158 (54.7%) outlets followed by consumer demand in 73 (25.5%) outlets. The government recommendation guided decision for restocking in both public in 125 (99.5%) and not-for-profit in 51 (76.7%) outlets while private outlets restocked depending on most profitable in 57 (59.2%) and consumer demand in 25 (26.3%) among other reasons.

### The price of anti-malarials

Anti-malarials in the public outlets were provided for free and were not included in the price analysis. Prices of anti-malarials with the same generic names were recorded and median prices were computed. The overall median prices for AL, other forms of ACT and quinine were USD 0.94 (R = 0.63-1.25), USD 5.63 (R = 1.88-8.13) and USD 1.25 (R = 0.75-1.25), respectively. The median price for all anti-malarials categories was much higher in private outlets than in not-for-profit outlets, as shown in Table 3. Other forms of ACT were more highly priced than the subsidized ACT and quinine.

**Table 3:** Median prices and Median Price Ratios of anti-malarials being sold in private and not-for-profit outlets

	**Outlet type**			
**Anti-malarials**	**Private**		**Not-for-profit**	
	**Median price (range) USD**	**Median price ratio**	**Median price (range) USD**	**Median price ratio**
AL*	0.94 (0.63-1.25)	1.88	0.75 (0.63-1.00)	1.5
Other forms of ACT**	6.0 (1.88-10.63)	12	2.5 (1.88-3.13)	5
Quinine*	1.0 (0.75-1.25)	1.33	0.75 (0.63-1.13)	1

USD, United States Dollars; ACT, artemisinin-based combination therapy; AL, artemether-lumefantrine.

*Median price ratio was calculated using median price of quinine in not-for-profit as reference since there is no government subsidized price.

**Median price ratio was calculated as anti-malarial median price to the government recommended price of subsidized ACT (AL) (KES 40, USD 0.5).

The median price ratio (MPR) for other forms of ACT was computed using the government subsidized price of ACT of USD 0.5. The private outlets had higher MPR for other forms of ACT compared to their not-for-profit counterparts (Table 3). Although AL was available in all the outlet types, the government recommended price of USD 0.5 did not apply and AL was sold at median price of USD 0.94 (R = 0.63-1.25) and USD 0.75 (R = 0.63-1.0) in private and not-for-profit outlets, respectively. Other forms of ACT were on average 12 times higher in price than AL in private outlets and five times higher in not-for-profit outlets (Table 3).

### Affordability of anti-malarials

All the anti-malarials were more affordable in not-for-profit outlets than private outlets. The quinine purchased within not-for-profit outlets was most affordable to household with lowest disposable income levels (Table 4). Other forms of ACT were least affordable and would cost up to 3.20 days of disposable income in private outlets.

**Table 4:** Number of days of disposable income to afford anti-malarials

	**Outlet type**	
	**Private**	**Not-for-profit**
**Anti-malarials**		
**AL**	0.50	0.40
**Other forms of ACT**	3.20	1.34
**Quinine**	0.54	0.40

AL, artemether-lumefantrine; ACT, artemisinin-based combination therapy. Affordability was expressed as median price (numerator) against lowest daily disposable income level (at KES 150, USD 1.875 = denominator).

## Discussion

The current study was designed to assess access to Kenya’s policy recommended anti-malarials, ACT and quinine in the public, private and not-for-profit drug outlets using the WHO and Healthcare Associated Infection (HAI) standardized methodologies for availability, prices and affordability of drugs. The findings of the current survey revealed that the government-recommended anti-malarials were available in all outlet types with public outlets providing only the two policy recommended anti-malarials; subsidized ACT (AL) and quinine. The private outlets on the other hand had stocked various other Artemisinin-based combinations in addition to the policy recommended anti-malarials. Stocking other forms of ACT in private outlets could be due to the fact that selling subsidized ACT would be unprofitable. Other studies in the region similar to the current study site (in terms of malaria endemicity) showed that private sectors prefer stocking drugs which can retail at competitive prices [26]. This is a matter of concern given that many households in the current study bought drugs from private outlets, where anti-malarials were sold at higher prices.

Low availability of all the four weight-specific packs of AL is of more concern, and worse still, the low availability of the six-tablet Pack meant for the treatment of under-fives, which is the most vulnerable group due to malaria infections. Coupled with the higher frequency of stock-outs recorded in this study, the calculation of appropriate regimen may not be guaranteed when the right package size is lacking, especially with low knowledge of providers on AL regimen in the private outlets as observed in a previous study in this region [27]. For example, giving four packets of the six-tablet pack for treatment of malaria in patients who need a 24-tablet pack or splitting the blister pack into 12-tablet pack for a child weighing 9 kg might result into uncertainty in the use of the drug as per its recommended regimen. Simplified packaging of anti-malarials has been found to improve the use of these drugs. For example, a previous study carried out in Uganda showed that patients’ adherence was high when they received AL blister packs with a weight-specific number of tablets and including pictorial instructions on how to use it [28]. This observation was also recorded in a study in the coastal region of Kenya, where simplicity of drug regimen was associated with increased treatment adherence [29].

The current study findings, consistent with previous ones [20] observed that lack of drugs in the formal sector contributes to people buying drugs from non-formal outlets. In such informal set-ups, the quality of drugs is less controlled and information on dose is not often provided. Yet in another study, about a third of individuals who sought care from public health facilities did not get drugs from the hospital pharmacy because they were out of stock [30]. The high stock-out rates questions the logic behind WHO’s call to treat malaria cases within 24 hours. Further questions are also raised on the recommendation to treat only laboratory-confirmed malaria cases, as well as confining ACT to formal sector providers, since these drugs are inaccessible especially in the rural areas, a part poorly covered by these facilities. Unless the communities are involved in the implementation of these guidelines, the current trend in malaria treatment in this region might start leaning towards the negative.

The prices of subsidized ACT remained lower than other anti-malarials in all the outlets. The lower median price in not-for-profit outlets was as a result of this sector being supplied with subsidized ACT by the government. In this sector, there was a full adherence to the subsidy policy as much as the anti-malarials were not free as in the public outlets. The not-for-profit outlets had additional service charge, further raising the price of the drugs. Lower prices of subsidized ACT are highly commendable, although it is not affordable to people who are of lowest economic levels. For instance, buying a full course of subsidized ACT would cost USD 0.94 in private and USD 0.75 in not-for-profit outlets, thus requiring up to 0.5 and 0.4 days of disposable household income, respectively. These observations from private outlets are consistent with previous data obtained from Kenya, Madagascar and Tanzania while the observed prices in the current study are lower than for Ghana, Zanzibar, Niger, Nigeria and Uganda in a recent multi-country independent evaluation survey in which the median prices ranged from USD 0.58 to USD 1.96 [31]. Conversely, the price of first-line treatment in the current study is higher than the price of first-line treatment in Burundi which was USD 0.16 in 2011 [26].

Higher prices than recommended in private outlets indicate lack of regulations and price control. This lack of control affects public health in the society, especially the most vulnerable groups to malaria-related morbidity and mortality due to poverty [32]. However, the price differences between the private and not-for-profit outlets for both subsidized ACT and quinine need to be reconsidered. Given the 3.2-fold price differences of other forms of ACT in the private outlets as compared to the not-for-profit outlets, there is need to lower these prices in the private outlets to make them comparable to those in other outlets. The reasons for reconciling and regulating prices may range from the fact that many households bought anti-malarials from private outlets due to frequent stock-outs in the public outlets. Secondly, the price is a vital factor in determining how drugs are used in the household. Thirdly, the private outlets are more accessible in terms of distance, as it has been previously demonstrated in a study in this region [16]. Considering the three reasons which show evidence of the role played by private sector in providing treatment for patients in western Kenya, it is important to effectively increase the affordability of other artemisinin-based combinations in these outlets. This strategy would further increase adherence to treatment regimen since the affected population would be able to afford the full course of anti-malarials. The end results would be among other reasons, preventing the irrational use of drugs, hence slowing down the development of resistance by malarial parasite. The observations in the current study fully support the WHO echoes that when health systems are not able to provide ACT at little or no cost, consumers may have to purchase them in the private sector [4].

## Conclusion

Availability of anti-malarials (ACT and quinine) still remains a challenge in the outlets in malaria endemic regions of western Kenya. First-line therapy for uncomplicated malaria, the subsidized ACT, was more available in public and not-for-profit facilities although with frequent stock-outs reported in public outlets. The prices remains high in the private outlets despite the fact that more households get their drugs from this sector hence making affordability a challenge. Private-sector marketing of ACT has resulted into use of sub-optimal doses because of partial sales of course of treatment packages. Collectively, availability, price and affordability of the drugs affect adherence to the dosing schedules during the treatment course.

## Recommendations

The study is in support of programmes that can support the subsidy policy and should be recommends that the programmes should be extended to include other anti-malarials to make them affordable in all sector outlets. In addition, frequent stock-outs of the required anti-malarials for different weight groups calls for more emphasis on the implementation of malaria treatment policy. It is highly advised that a creation of private-public partnership in the procurement and distribution of recommended anti-malaria drugs is the goal to make them available for the customers in all the outlets. The factors that influence availability should be tackled according to regional context. These should include monitoring net sales/consumption rates through inventory, time frame estimates and physical stock recording, especially according to seasonality of illness and differences in district health needs. Internal agreement to transfer anti-malarials from one sector outlet to another and from one region to another depending on the disease burden and consumption rate is proposed.

The government needs to implement price regulatory policies to protect the most vulnerable groups from exploitation by establishing effective surveillance mechanisms. The prices should also be regulated to ensure uniformity in pricing of the same product in all sector outlets. Finally, appropriate health promotion programmes on the importance of anti-malarial adherence can be made available to the community. This could be done, for example, through pamphlets in medical facilities, regular public service announcements and mass media including use of television, radio and video shows or through a more community-focused method. Following a switch in national drug policy to ACT, behaviour change communication is recommended during which all outlets staff should be trained in unbiased manner on the current malaria policy to improve drug use in the community. In areas where such interventions have been performed, then further scaling up of these programmes is a necessity.

## Competing interests

There is no competing interest from any of the authors of the manuscript due to commercial or other affiliations.

## Authors’ contribution

CAW designed, carried out the survey studies in the drug outlets and households. CAW and CO performed the statistical analysis and participated in drafting the manuscript. All authors read and approved the final manuscript.
